# Dropped head syndrome as the sole presenting manifestation of probable post-cytomegalovirus immune-mediated brainstem encephalitis in an immunocompetent woman: a case report

**DOI:** 10.3389/fimmu.2026.1861881

**Published:** 2026-08-28

**Authors:** Bo Zhou, Zhibo Song, Ni Bi, Tao Li, Li Zhang, Zhi Zhao, Yingsong Wang

**Affiliations:** Department of Orthopedics, The Second Affiliated Hospital of Kunming Medical University, Kunming, China

**Keywords:** brainstem encephalitis, case report, corticosteroid therapy, cytomegalovirus, dropped head syndrome, multidisciplinary diagnosis, post-infectious autoimmunity

## Abstract

**Background:**

Dropped head syndrome (DHS) is a rare, disabling chin-on-chest deformity caused by severe cervical extensor weakness. The etiologic spectrum is dominated by peripheral myopathies, motor neuron disease, and myasthenia gravis; central nervous system causes are seldom considered, particularly in older adults where coexisting cervical spondylosis appears to offer a unifying structural explanation. To our knowledge, DHS as the sole presenting manifestation of brainstem encephalitis has not been previously reported.

**Case presentation:**

A 60-year-old immunocompetent woman presented with four months of progressive cervical kyphosis beginning two weeks after a self-limited upper respiratory illness. Initial orthopedic evaluation attributed her posture to degenerative cervical disease, but the structural findings did not account for the severity of selective neck-extensor weakness. Multidisciplinary reassessment disclosed bilaterally absent gag reflexes and rightward tongue deviation, cranial-nerve signs that localized dysfunction to the medulla. Lumbar puncture showed mildly elevated protein (0.73 g/L) and a distinctive intrathecal immunoglobulin pattern, with markedly elevated IgA and IgM and mildly elevated IgG. CSF was positive for cytomegalovirus (CMV)–specific IgG alone among the TORCH antigens tested, despite serum positivity for CMV, herpes simplex virus type 1, and rubella; CMV DNA by PCR was negative. Paired quantitative titres yielded a CSF antibody-specific index of approximately 10, consistent with intrathecal CMV-specific antibody synthesis. Contrast-enhanced (gadobutrol) and STIR craniocervical MRI, together with brain MRI, showed no abnormal cord or brainstem enhancement or acute signal abnormality. Competing etiologies—structural cervical disease, primary and hypothyroid myopathy, CNS tuberculosis, and paraneoplastic syndrome—were systematically excluded. A 14-day course of moderate-dose corticosteroids, combined with cervical orthosis and structured rehabilitation, produced complete resolution. Remission was sustained at one-year follow-up; surgery was avoided.

**Conclusions:**

DHS can be the sole presenting manifestation of probable post-CMV immune-mediated brainstem encephalitis, even when brain MRI is unrevealing and cervical degeneration appears to provide a structural explanation. Careful cranial-nerve examination, CSF immunological profiling with intrathecal immunoglobulin quantification, and multidisciplinary reassessment are essential for distinguishing central from peripheral DHS. In this patient, moderate-dose corticosteroids produced complete recovery even when initiated well outside the conventional early treatment window, and potentially obviated cervical surgery.

## Introduction

Dropped head syndrome (DHS) is a passively correctable chin-on-chest deformity caused by severe cervical extensor weakness. Most reported cases stem from peripheral neuromuscular disorders (1, 2); central nervous system (CNS) etiologies are rarely considered (3). This bias has particular consequences in older adults. Age-related cervical spondylosis is near-universal, and routine magnetic resonance imaging (MRI) almost always reveals an age-appropriate lesion that appears to fit the clinical picture. Attributing the deformity to these findings may lead to cervical fusion for a condition that surgery cannot correct (4).

Upright head control depends on tonic descending input from the brainstem. Focal neuroinflammation can disrupt this input without producing fever, encephalopathy, or long-tract signs. Cytomegalovirus (CMV) illustrates how this may occur. Symptomatic CMV disease of the CNS is uncommon in immunocompetent adults but is likely under-recognised (5). More relevant to the present case, a self-limited CMV infection can be followed by immune-mediated inflammation of the CNS after the acute illness has resolved. In that setting, CSF CMV-specific IgG with a negative CMV PCR signals immune-mediated injury rather than active replication (6, 7). Post-viral CNS autoimmunity is increasingly framed in terms of compartmentalised intrathecal immune activation, driven by cytokine milieu, pattern-recognition receptors, and T- and B-cell responses rather than ongoing viral lysis (8). When early inflammation escapes non-contrast MRI, the resulting clinico-radiological dissociation conceals the diagnosis.

The distinction between central and peripheral causes matters, because a central inflammatory cause may be reversible with immunotherapy. We report a 60-year-old immunocompetent woman with isolated DHS attributed to brainstem-MRI–negative, CMV-triggered immune-mediated rhombencephalitis. Moderate-dose corticosteroids initiated four months after onset, combined with rehabilitation, produced complete resolution and averted surgery. The case bears on three questions: how to distinguish central from peripheral DHS when cervical degeneration confounds the picture; what mechanism may underlie MRI-negative presentations; and whether immunotherapy retains efficacy outside the early treatment window.

## Case presentation

### Presenting concerns

A 60-year-old immunocompetent woman was admitted for four months of progressive inability to hold her head upright. Symptoms developed insidiously after a self-limited upper respiratory illness two weeks earlier, marked by transient dysphagia and hoarseness that resolved with oral antibiotics; the cervical extensor weakness persisted and worsened. Outside evaluation had attributed the deformity to cervical spondylosis on CT and offered no specific treatment. Her history included poorly controlled hypertension for more than ten years, no prior surgery, malignancy, neurological disease, or immunocompromise, and no immunosuppressive medication.

### Clinical findings on admission

**Figure 1:**
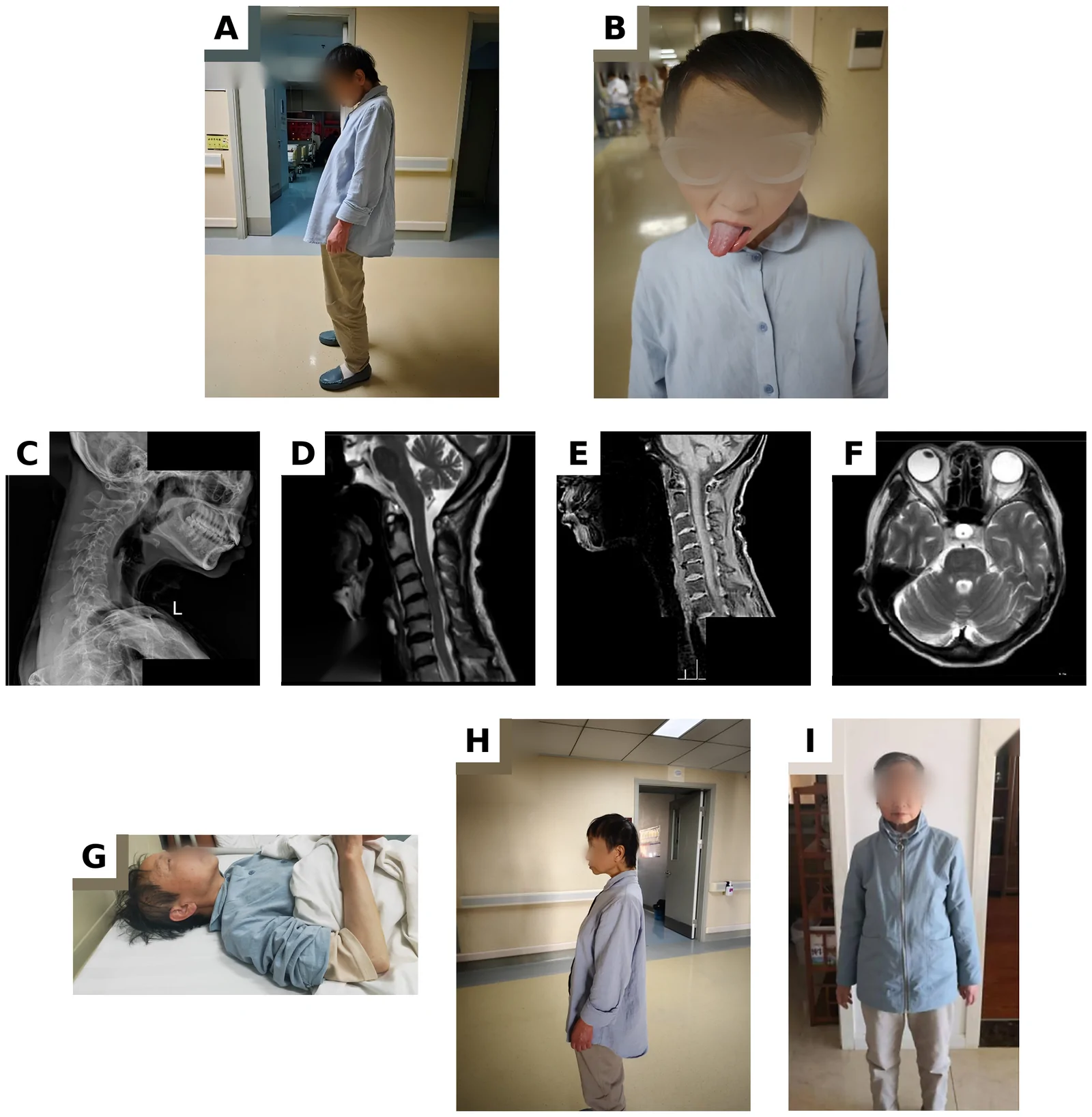
Clinical course from admission to one-year follow-up. **(A)** Admission: severe chin-on-chest deformity on standing. **(B)** Admission: rightward deviation of the tongue on protrusion, consistent with left hypoglossal-nucleus dysfunction. **(C)** Cervical radiograph (lateral): kyphosis with C2–C3 anterior translation and multilevel degenerative change. **(D)** T2-weighted sagittal cervical MRI: multilevel disc protrusion, C3–C7 canal stenosis with mild cord indentation. **(E)** Contrast-enhanced (gadobutrol) T1-weighted sagittal image of the craniocervical junction: no abnormal enhancement of the lower brainstem or cervical cord. **(F)** T2-weighted axial brain MRI at the level of the pons: no acute brainstem parenchymal signal abnormality. **(G)** Hospital day 9: able to lie supine without pillow support, indicating the earliest recovery of tonic neck-extensor activity. **(H)** Hospital day 11: erect standing posture with full active cervical extension [compare with **(A)**]. **(I)** One-year outpatient follow-up: complete resolution of cervical kyphosis with sustained upright posture and no recurrent deformity. Scale bars are not applicable for clinical photography.

Examination showed a passively correctable chin-on-chest deformity with severely limited active cervical extension in both standing and sitting positions (**Figure 1A**). The posterior cervical muscles were atrophic. The remainder of the spine was unremarkable.

Initial neurological examination by the orthopedic team documented 5/5 limb power, normal bulk and tone, intact sensation, and no ataxia. Deep tendon reflexes were diffusely brisk bilaterally; Babinski, Gordon, Oppenheim, and Hoffmann signs were negative; there was no meningismus. No cranial-nerve abnormality was identified.

On hospital day 4, prompted by discordant imaging and laboratory findings (detailed below), neurological reassessment disclosed two new findings: rightward deviation of the tongue on protrusion (**Figure 1B**) and bilaterally absent gag reflexes. Speech was clear; ocular movements, facial strength, and hearing were preserved. The combination of selective neck-extensor weakness, tongue deviation, and absent gag reflexes localized the lesion to the medulla—implicating the hypoglossal and glossopharyngeal nuclei—and shifted the diagnostic direction from structural cervical disease toward a central process.

### Diagnostic assessment

#### Laboratory investigations

Admission workup (**Supplementary Table 1**) disclosed four findings relevant to the differential diagnosis: overt hypothyroidism (free T4 9.34 pmol/L; TSH 7.49 mIU/L), mildly elevated creatine kinase (332 U/L) with myocardial isoenzyme fraction within the expected proportion, a marked immunological disturbance (rheumatoid factor IgM 93.3 IU/mL; serum IgA 5.27 g/L; reduced complement C3), and a positive T-SPOT.TB interpreted as latent infection. Antinuclear and anti–double-stranded DNA antibodies were negative. Serum TORCH screening showed IgG positivity for CMV, HSV-1, and rubella consistent with prior exposure. Paraneoplastic screening was negative.

Lumbar puncture on day 4 (**Table 1**) showed normal opening pressure and a mildly elevated CSF protein (0.73 g/L; ref 0.15–0.45). The intrathecal immunoglobulin profile was distinctive: IgG was only mildly elevated (66.0 mg/L; 1.1× upper reference limit), whereas IgA (17.80 mg/L) and IgM (1.52 mg/L) were markedly elevated—approximately 9-fold and 5-fold above their upper reference limits. CSF TORCH IgG was selectively positive for CMV alone, despite serum positivity for CMV, HSV-1, and rubella; CSF CMV DNA by PCR was negative. Paired serology gave a CSF CMV-IgG titre of 114.9 U/mL against a serum titre exceeding 2000 U/mL; with CSF and serum total IgG of 66.0 mg/L and 11.94 g/L, the calculated CSF antibody-specific index was approximately 10, well above the threshold of 1.5 taken to indicate intrathecal synthesis. CSF was negative for rubella- and HSV-1-specific IgG despite serum seropositivity, serving as internal negative controls. Selective CSF reactivity to CMV in the absence of detectable viral DNA, together with marked intrathecal IgA and IgM elevation, pointed to immune-mediated CNS inflammation following prior CMV exposure rather than active replication.

**Table 1:** Cerebrospinal fluid analysis (performed on hospital day 4).

Parameter	Result	Reference range	Interpretation
Opening pressure	Normal	—	—
Appearance	Clear, colorless	—	—
Total protein (g/L)	0.73 ↑	0.15–0.45	Elevated
Glucose	Within normal limits	—	—
Cell count and differential	Within normal limits	—	—
Intrathecal immunoglobulins			
CSF IgG (mg/L)	66.0 ↑	4.8–58.6	~1.1× ULN
CSF IgA (mg/L)	17.80 ↑↑	<2	~9× ULN
CSF IgM (mg/L)	1.52 ↑↑	0.19–0.29	~5× ULN
Virological studies			
CSF CMV-specific IgG	Positive (qualitative)	—	Selective intrathecal reactivity to CMV
CSF HSV-1 IgG	Negative	—	—
CSF rubella IgG	Negative	—	—
CSF CMV DNA (PCR)	Negative	—	Non-replicative
Mycobacterial studies			
Acid-fast bacilli smear	Negative	—	CNS-TB excluded
Adenosine deaminase activity	Non-elevated	—	CNS-TB excluded

CMV, cytomegalovirus; CSF, cerebrospinal fluid; CNS, central nervous system; HSV-1, herpes simplex virus type 1; PCR, polymerase chain reaction; TB, tuberculosis; ULN, upper limit of normal.↑ indicates a value above the upper limit of the reference range, and ↑↑ indicates a value markedly above the upper limit of the reference range (more than fivefold).

#### Electrophysiology

Two electrodiagnostic studies (performed two days apart) were concordant (**Supplementary Table 2**). Motor and sensory nerve-conduction studies, F-wave latencies, and sympathetic skin responses were normal. Needle electromyography showed no fibrillation potentials or positive sharp waves; short-duration motor-unit potentials with early recruitment were recorded in the bilateral C5–C6 cervical paraspinal muscles, with subtler short-duration potentials in the left triceps brachii, left extensor digitorum communis, and left rectus femoris. These limb findings were clinically silent (limb power 5/5). In the absence of active denervation, with normal nerve conduction and coexisting cranial-nerve signs, this pseudomyopathic pattern is best interpreted as secondary motor-unit remodelling rather than a primary or inflammatory myopathy. Repetitive nerve stimulation was not performed.

#### Imaging

Full-spine standing radiographs (**Supplementary Figure 1**) showed cervical kyphosis and mild left-sided cervicothoracic scoliosis without significant thoracic or lumbar deformity. Cervical imaging confirmed multilevel degeneration with C2–C7 disc protrusions, C3–C7 central canal stenosis with mild cord indentation, C5–C6 disc-space narrowing, and interspinous ligament edema at C2–C7 (**Figures 1C, D**). Dynamic films showed C2–C4 instability with mild anterior translation of C2 and C3. The degree of cord compression did not match the severity or selectivity of the cervical extensor weakness.

Brain MRI demonstrated chronic microangiopathic white-matter disease (Fazekas grade 3) and an old right temporal hemorrhagic lesion; diffusion-weighted imaging showed no acute infarct. Contrast-enhanced (gadobutrol) and fat-suppressed (STIR) sequences of the cervical spine and craniocervical junction, extending to the lower brainstem and cervical cord, showed no abnormal enhancement or cord signal change; a dedicated high-resolution contrast-enhanced brainstem protocol was not separately obtained. Critically, no acute parenchymal signal abnormality, enhancement, or mass effect was identified within the brainstem (**Figures 1E, F**). Cardiac workup (echocardiography and coronary CT angiography) showed mild mitral and tricuspid regurgitation and a non-calcified left anterior descending artery plaque (CAD-RADS 2/P1).

#### Multidisciplinary team consultation

By hospital day 4 the clinico-radiological dissociation prompted formal MDT discussion involving neurology, neurosurgery, rheumatology, and neurorehabilitation. Targeted re-examination disclosed the previously unrecognized cranial-nerve signs and prompted lumbar puncture. Rheumatology judged rheumatic disease unlikely. Neurorehabilitation proposed post-viral brainstem encephalitis as the leading differential. Synthesizing the clinical picture (selective neck-extensor weakness with bulbar signs), electrophysiology (paraspinal myopathic pattern without peripheral nerve involvement), CSF findings (protein elevation with marked intrathecal IgA and IgM rise; selective CSF CMV-IgG; negative CMV PCR), and imaging (no acute brainstem parenchymal change), and having excluded structural cervical disease, primary myopathy, hypothyroid myopathy (see Discussion), CNS tuberculosis, and paraneoplastic syndrome, the working diagnosis was post-CMV immune-mediated brainstem encephalitis presenting as isolated DHS.

### Therapeutic intervention

Empirical immunomodulation was initiated (timeline in **Supplementary Table 3**). Given the patient’s age and cardiovascular comorbidities, a moderate-dose regimen was selected over high-dose pulse therapy to minimize the risks of hyperglycemia, infection, and osteoporotic fracture (9). Intravenous dexamethasone 10 mg daily was given for 7 days, followed by oral prednisone 40 mg daily for a further 7 days, then discontinued without taper. No antiviral therapy, intravenous immunoglobulin, or plasma exchange was administered. Structured rehabilitation began simultaneously: physiotherapist-supervised neck-extensor strengthening, postural retraining, and a rigid cervical collar during upright activities.

### Follow-up and outcomes

Clinical response was rapid. By hospital day 9 the patient could lie supine without pillow support, indicating recovery of resting neck-extensor tone (**Figure 1G**). By day 11 she had regained full active cervical extension standing, walked with the head erect, and resumed basic activities of daily living (**Figure 1H**); tongue deviation had diminished markedly. She was discharged on day 14 with complete resolution of the deformity. At one-year follow-up she had returned to full activities of daily living with no recurrence, no new neurological findings, and complete preservation of the correction achieved during admission (**Figure 1I**). No further immunosuppression had been required, and no relapse had occurred.

### Patient perspective

The patient reported that recovery of head posture, after more than four months of progressive disability, restored her confidence and social participation. She particularly valued the multidisciplinary decision that avoided the cervical fusion initially contemplated. She has consented to publication of this report, including clinical photographs, in the interest of improving recognition of this presentation.

## Discussion

**Figure 2:**
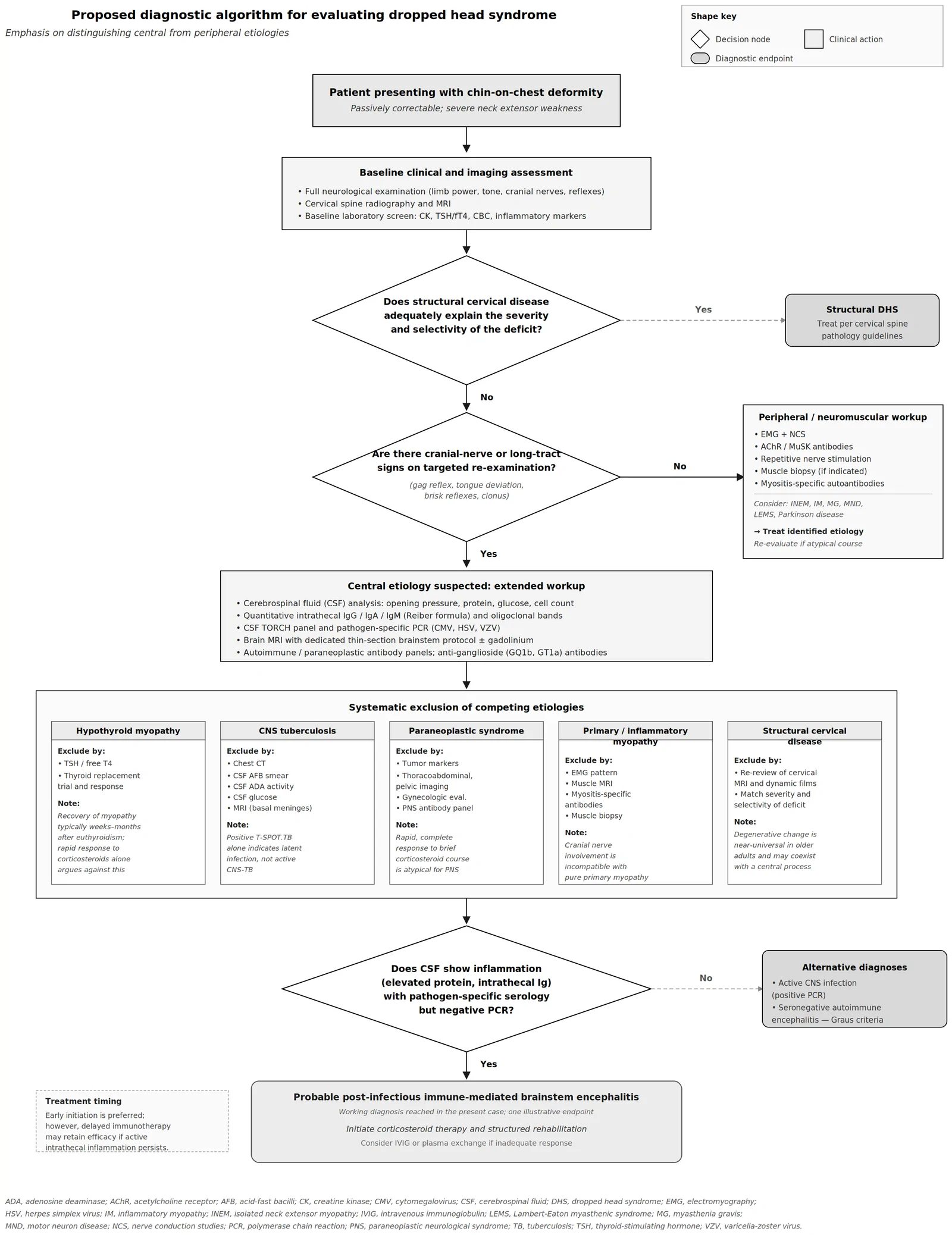
Proposed diagnostic algorithm for evaluating dropped head syndrome, with emphasis on distinguishing central from peripheral etiologies. Initial evaluation comprises neurological examination, cervical imaging, and baseline laboratory screening. The first decision point assesses whether structural cervical disease adequately explains the severity and selectivity of the deficit; when it does not, targeted re-examination for cranial-nerve and long-tract signs should follow. A positive finding triggers extended central-nervous-system workup, including cerebrospinal fluid analysis with intrathecal immunoglobulin profiling, pathogen-specific serology, and dedicated brainstem imaging. Systematic exclusion of competing etiologies (hypothyroid myopathy, central-nervous-system tuberculosis, paraneoplastic syndromes, primary or inflammatory myopathy, and structural cervical disease) is performed in parallel. The convergence of intrathecal inflammation, pathogen-specific CSF antibody positivity, and negative polymerase chain reaction supports a working diagnosis of post-infectious immune-mediated brainstem encephalitis. The algorithm is intended as a general framework for evaluating dropped head syndrome and for separating central from peripheral causes; the specific investigations shown are prompted by the clinical phenotype and neurological findings rather than applied to every patient, and the post-infectious pathway is one illustrative endpoint. Dashed arrows indicate alternative diagnostic pathways. ADA, adenosine deaminase; AChR, acetylcholine receptor; AFB, acid-fast bacilli; CK, creatine kinase; CMV, cytomegalovirus; CSF, cerebrospinal fluid; DHS, dropped head syndrome; EMG, electromyography; HSV, herpes simplex virus; IM, inflammatory myopathy; INEM, isolated neck extensor myopathy; IVIG, intravenous immunoglobulin; LEMS, Lambert-Eaton myasthenic syndrome; MG, myasthenia gravis; MND, motor neuron disease; NCS, nerve conduction studies; PCR, polymerase chain reaction; PNS, paraneoplastic neurological syndrome; TB, tuberculosis; TSH, thyroid-stimulating hormone; VZV, varicella-zoster virus.

We report a 60-year-old immunocompetent woman in whom isolated DHS proved to be the presenting feature of post-CMV immune-mediated brainstem encephalitis. Three aspects merit emphasis. The brainstem localization emerged only when multidisciplinary reassessment elicited cranial-nerve signs missed at admission. The diagnosis became clear only after several competing explanations had been considered in turn. Cervical spondylosis with mild cord compression, overt hypothyroidism, latent tuberculous exposure, and a myopathic-pattern electromyogram could each account for part of the presentation, and each was excluded on the grounds set out below. Chronic microangiopathic white-matter disease (Fazekas grade 3) and an old right temporal hemorrhagic lesion were also present on brain MRI, but neither can cause selective neck-extensor weakness with lower cranial-nerve signs, and both were regarded as incidental. Full recovery followed 14 days of moderate-dose corticosteroids and rehabilitation begun more than four months after symptom onset, without pulse therapy and without surgery (**Figure 2**).

The decisive step in this case was a re-examination rather than a new investigation. Structural cervical disease was present, but limb power, sensation, and long-tract signs were preserved, and the degree of compression was too mild for a deficit of this severity. This clinico-radiological dissociation triggered multidisciplinary review, at which bilaterally absent gag reflexes and rightward tongue deviation localized dysfunction to the glossopharyngeal and hypoglossal nuclei. Neither sign had been recorded at admission. When isolated axial weakness is attributed to structural cervical disease, focused neurological re-examination should therefore precede any surgical decision.

We found no comparable report. A search of PubMed, Web of Science, and Embase (inception to August 2026), combining “dropped head syndrome,” “camptocephalia,” and “chin-on-chest” with “encephalitis,” “rhombencephalitis,” “brainstem,” and “cytomegalovirus,” returned no case in the English-language literature in which DHS was the sole presenting manifestation of brainstem encephalitis. The nearest reports differ in an important respect. CMV encephalomyelitis in an immunocompetent adult has been described with a biphasic viral and then immune-mediated course, but with widespread neuraxial involvement rather than isolated axial weakness (7). Post-infectious CMV rhombencephalitis has been reported with the expected brainstem syndrome of ataxia, ophthalmoplegia, and altered consciousness (6). In our patient there was no accompanying brainstem syndrome, and the head drop was the only manifestation.

The differential diagnosis for DHS in older adults is broad. Structural cervical disease was present but could not account for the cranial-nerve signs or the severity of the cervical extensor weakness, and was therefore insufficient as a unifying diagnosis. Isolated neck extensor myopathy and inflammatory myopathy were considered. Electromyography showed short-duration, early-recruitment motor units in cervical paraspinal muscles and, to a lesser degree, in selected limb muscles, but the limb findings were clinically silent, and cranial-nerve involvement is incompatible with a pure primary myopathy (10). This paraspinal signature is best interpreted as a pseudomyopathic pattern—motor-unit remodelling secondary to sustained axial mechanical disadvantage and central disinnervation rather than intrinsic muscle-fibre pathology (3).

Anti-MuSK and anti-acetylcholine-receptor myasthenia gravis deserve specific consideration, since both can present with predominant neck-extensor weakness and bulbar features (11, 12). We did not measure serum acetylcholine-receptor or MuSK antibodies and did not perform repetitive nerve stimulation, so a neuromuscular-junction disorder cannot be excluded on electrophysiological grounds. Several features nonetheless argue against it. The weakness showed no fatigability or diurnal variation and spared the extraocular and levator palpebrae muscles, nerve conduction and F-waves were normal without active denervation, and complete recovery followed only 14 days of moderate-dose corticosteroids, with no acetylcholinesterase inhibitor or maintenance immunotherapy and sustained drug-free remission at one year. This course fits poorly with the chronic, fluctuating and treatment-dependent nature of myasthenia gravis.

Hypothyroid myopathy warranted particular attention, as the patient had overt biochemical hypothyroidism (low free T4, elevated TSH), and hypothyroidism is a recognized cause of proximal and axial myopathy. One observation argues against it as the driver: thyroid hormone replacement was not initiated, yet cervical extensor function was fully restored within two weeks of corticosteroid administration. Hypothyroid myopathy responds to thyroid replacement rather than corticosteroids, and clinical recovery typically requires weeks to months even after biochemical euthyroidism is achieved (13).

Active tuberculosis and tuberculous meningoencephalitis were excluded on the basis of normal chest CT, negative CSF acid-fast bacilli smear, non-elevated CSF adenosine deaminase, normal CSF glucose, and absence of basilar meningeal enhancement. The positive T-SPOT.TB was therefore interpreted as latent infection rather than active disease (14). Paraneoplastic syndrome was considered given the patient’s age and subacute course; systematic tumor screening was negative. Formal paraneoplastic antibody panels were not obtained, but the absence of malignancy combined with rapid, complete response to brief corticosteroid monotherapy argues against a paraneoplastic process, which characteristically responds only partially or with delay to first-line immunotherapy (15).

Several positive findings converged on an immune-mediated brainstem process associated with prior CMV exposure. The CSF was not bland: protein was mildly elevated at 0.73 g/L, and quantitative intrathecal immunoglobulins showed a distinctive pattern—mild IgG elevation (66 mg/L) with markedly elevated IgA (~9-fold above the upper reference limit) and IgM (~5-fold), indicating compartmentalized intrathecal immune activation rather than simple blood–brain barrier disruption (16–18). Virological testing was also informative. Of three viruses with positive serum IgG (CMV, HSV-1, rubella), only CMV-specific IgG was detectable in CSF, and CMV DNA by PCR was negative. The selective CSF reactivity to CMV—despite serum positivity for all three viruses—supports intrathecal synthesis specific to CMV rather than passive transudation through a disrupted blood–brain barrier. These qualitative observations can now be formalised. CSF CMV-IgG was 114.9 U/mL against a serum titre exceeding 2000 U/mL; with CSF and serum total IgG of 66.0 mg/L and 11.94 g/L, the CSF antibody-specific index was approximately 10, well above the 1.5 threshold conventionally taken to indicate intrathecal synthesis. CSF was negative for rubella- and HSV-1-specific IgG despite serum seropositivity, internal negative controls that argue against passive transudation. Because CSF albumin was not measured, this index was normalised to total IgG rather than to an albumin quotient, and the serum titre at assay ceiling makes it a conservative estimate; together these findings point to an immune-mediated process following prior CMV exposure rather than active replication (6, 7).

The therapeutic response offers further indirect support. Under the Graus 2016 clinical framework, subacute onset of focal CNS dysfunction, new CSF inflammatory findings without identified autoantibodies, and reasonable exclusion of alternative causes are taken as meeting the criterion of antibody-negative but probable autoimmune encephalitis, with response to immunotherapy incorporated as a supporting diagnostic pillar (19). We recognise that our patient’s brainstem-restricted presentation sits at the interface between this framework and the narrower category of post-infectious rhombencephalitis and does not map cleanly onto any single published case-definition. Meaningful improvement appeared within one week of corticosteroid initiation, and cervical extensor weakness resolved completely by the end of the second week. The speed and completeness of response to corticosteroid monotherapy—without concurrent antiviral therapy—fits suppression of immune-mediated inflammation better than control of active viral replication.

Several mechanistic frameworks for post-infectious autoimmune rhombencephalitis have been advanced (8), including molecular mimicry targeting neuronal gangliosides (as in Bickerstaff brainstem encephalitis) (20) and T-cell–mediated rhombencephalitis (21). We did not obtain the antibody or cellular panels required to adjudicate between these possibilities and therefore do not extend the mechanistic claim beyond the general framework of post-infectious immune-mediated brainstem inflammation. The patient’s wider immunological profile—elevated rheumatoid factor, reduced complement C3, elevated serum IgA, biochemical hypothyroidism, and latent tuberculous exposure—suggests a background of subclinical immune dysregulation that may have lowered the threshold for post-viral immune-mediated injury. Each of these findings carries a non-trivial baseline prevalence in older adults, so this observation should be regarded as generating rather than confirming a hypothesis.

A third framework, latent CMV reactivation against a backdrop of age-related immune remodelling, warrants specific consideration in this case. We raise it as a speculative backdrop rather than an established mechanism in this patient. Latent CMV infection of lymphatic endothelial cells can sustain CD8^+^ T-cell memory inflation and a chronic low-grade inflammatory state without productive viral replication (22)—a condition compatible with the negative CSF CMV PCR observed here. In older adults this process converges with broader patterns of CMV-associated immunosenescence and inflammaging (23, 24), which may lower the threshold for post-viral immune-mediated CNS injury. The CSF signature in our patient—selective intrathecal CMV-IgG positivity, disproportionate IgA and IgM elevation without IgG dominance, and absent viral DNA—fits compartmentalised immune activation triggered by prior CMV exposure rather than ongoing lytic infection (17). The rapid and complete response to corticosteroid monotherapy is consistent with suppression of glucocorticoid-responsive immune effectors (25) rather than antiviral control. We present this framework as hypothesis-generating; definitive confirmation would require cellular immunophenotyping and CMV-specific T-cell assays that were not performed in this case.

Two therapeutic observations in this patient may be of practical interest. Complete clinical recovery was achieved with moderate-dose corticosteroids—intravenous dexamethasone 10 mg daily for 7 days followed by oral prednisone 40 mg daily for approximately 7 days—rather than the high-dose pulse regimens more commonly used in autoimmune encephalitis (26). In elderly patients, pulse regimens carry disproportionate risk of hyperglycemia, infection, and osteoporotic fracture (9). That a moderate-dose regimen produced complete response in this patient deserves consideration, though generalization from a single case is not warranted. The total corticosteroid exposure was brief, and sustained remission at one year without relapse suggests the underlying inflammatory driver was suppressible with limited immunosuppression rather than a chronic, self-perpetuating autoimmune encephalitis requiring prolonged therapy.

Therapy was initiated more than four months after symptom onset—well outside the early treatment window conventionally emphasized in post-infectious and autoimmune encephalitides (27)—yet the response was rapid and complete. This observation aligns with a small but growing body of reports suggesting that some post-infectious brainstem encephalitides retain responsiveness to immunotherapy at later time points, particularly when active intrathecal inflammation persists (28). The CSF findings in our patient—mildly elevated protein and markedly elevated intrathecal IgA and IgM four months after symptom onset—indicate exactly this pattern of sustained intrathecal inflammation, offering a biological rationale for the late treatment response. It should not be taken as justification for treatment delay when earlier intervention is feasible.

Concurrent rigid cervical orthosis and physiotherapist-led extensor strengthening were introduced at corticosteroid initiation. The orthosis reduces sustained eccentric loading on weakened extensors and may create mechanical conditions permissive to functional recovery. In this framework, immunomodulation addresses the inflammatory driver and rehabilitation supports restoration of motor function.Several limitations deserve acknowledgment. This is a single case, and the diagnostic and therapeutic observations cannot be generalized without confirmation in larger series. Our etiologic workup did not include anti-ganglioside, autoimmune encephalitis, or formal paraneoplastic antibody panels, nor CSF oligoclonal band analysis; serum acetylcholine-receptor and muscle-specific kinase (MuSK) antibodies and repetitive nerve stimulation were not obtained, so although the sustained drug-free remission argues strongly against myasthenia gravis a neuromuscular-junction disorder cannot be formally excluded electrophysiologically; seronegative paraneoplastic presentations and autoantibody-positive forms responsive to corticosteroids (e.g., LGI1) cannot be formally excluded, although absence of malignancy at one-year follow-up argues against occult paraneoplasia. Although contrast-enhanced and STIR craniocervical sequences covering the lower brainstem were unremarkable, dedicated high-resolution brainstem protocols, diffusion tensor imaging and FDG-PET were not obtained; CSF albumin was not measured, so the antibody-specific index was normalised to total IgG rather than to an albumin quotient. A single CSF CMV PCR cannot fully exclude earlier transient replication; detectable CSF CMV-IgG supports prior exposure but does not by itself establish causation. Limb EMG changes were clinically silent, and muscle biopsy was not performed to distinguish subclinical myopathy from secondary neurogenic change. Follow-up beyond one year is needed to exclude late relapse, reported in some autoantibody-associated encephalitides at 1–3 years. Prospective DHS cohorts incorporating systematic CSF immunological profiling and targeted brainstem neuroimaging would help clarify the prevalence of central etiologies and refine the diagnostic approach to this under-recognized entity.

## Conclusion

This case shows that DHS can present as the sole manifestation of post-CMV immune-mediated brainstem encephalitis in an immunocompetent adult, and that coexisting cervical degeneration may obscure the central process. Three practice implications follow. First, any older adult with isolated DHS warrants a focused cranial-nerve and long-tract examination, even when cervical imaging offers a unifying structural diagnosis. Second, when clinico-radiological dissociation exists, CSF analysis incorporating quantitative intrathecal immunoglobulin profiling and pathogen-specific serology—not protein and cell count alone—can materially alter the diagnostic formulation. Third, moderate-dose corticosteroids with structured rehabilitation may achieve complete recovery even when initiated well outside the conventional early treatment window, potentially obviating major cervical reconstructive surgery.

## Data Availability

The original contributions presented in the study are included in the article/**Supplementary Material**. Further inquiries can be directed to the corresponding author.
