# Supplementary material for: Dropped head syndrome as the sole presenting manifestation of probable post-cytomegalovirus immune-mediated brainstem encephalitis in an immunocompetent woman: a case report
Source: Front Immunol. 2026 Aug 28;17:1861881. doi: 10.3389/fimmu.2026.1861881 (PMC13561995; doi:10.3389/fimmu.2026.1861881)
Supplement: Supplementary file 1 [file SupplementaryFile1.docx]

**Supplementary Materials**

*Dropped Head Syndrome as the Sole Presenting Manifestation of Post-Cytomegalovirus Immune-Mediated Brainstem Encephalitis in an Immunocompetent Woman*

**Contents**

Table S1. Laboratory findings on admission.

Table S2. Treatment timeline and clinical response.

Table S3. Electrodiagnostic findings (two concordant studies).

Supplementary Figure S1. Full-spine standing radiographs on admission (PA and lateral projections).

**Table S1. Laboratory findings on admission.**

| **Parameter** | **Result** | **Reference range** |
| --- | --- | --- |
| *Hematology* | | |
| Monocytes (%) | 12.3 ↑ | 3.0–10.0 |
| Lymphocytes (× 10⁹/L) | 1.08 (low-normal) | 1.1–3.2 |
| *Biochemistry* | | |
| Creatine kinase (U/L) | 332 ↑ | 26–140 |
| CK-MB (U/L) | 69 ↑ (range 66–73 over serial testing) | <25 |
| Aspartate aminotransferase (U/L) | 47 ↑ (range 45–49 over serial testing) | 8–40 |
| Lactate dehydrogenase (U/L) | 252 borderline ↑ | 109–245 |
| *Thyroid function* | | |
| TSH (mIU/L) | 7.49 ↑ | 0.27–4.2 |
| Free T4 (pmol/L) | 9.34 ↓ | 10–22 |
| Total T4 (nmol/L) | 40.17 ↓ | 50–130 |
| *Immunology* | | |
| Serum IgA (g/L) | 5.27 ↑ | 0.7–4.0 |
| Complement C3 (g/L) | 0.89 ↓ | 0.9–1.8 |
| Rheumatoid factor IgM (IU/mL) | 93.3 ↑↑ | <20 |
| C-reactive protein | Within normal limits | — |
| Antinuclear antibody | Negative | — |
| Anti–dsDNA | Negative | — |
| *Infectious screening* | | |
| T-SPOT.TB | Positive (latent) | — |
| HIV serology | Negative | — |
| *Serum TORCH (IgG)* | | |
| CMV IgG | Positive (prior exposure) | — |
| HSV-1 IgG | Positive (prior exposure) | — |
| Rubella IgG | Positive (prior exposure) | — |
| Myositis-specific antibody panel (Jo-1, PM-Scl, Ro-52, etc.) | Negative | — |
| ANCA (c-ANCA / p-ANCA) | Negative | — |
| Anti-glomerular basement membrane antibody | Negative | — |
| Antiphospholipid antibodies (anticardiolipin, anti-β2-glycoprotein I) | Negative | — |
| HLA-B27 | Negative | — |
| PNH screen (CD55/CD59) | Normal | — |
| Thyroid autoantibodies (TgAb, TPOAb, TRAb) | Negative | — |
| Serum CMV IgG, quantitative | >2000 U/mL (positive) | 0–25 |
| Serum rubella IgG, quantitative | 41.1 (positive) | 0–10 |
| Serum HSV-1 IgG, quantitative | 93.7 (positive) | <20 |
| *Tumor screening* | | |
| Tumor markers panel | All within normal limits | — |
| Imaging + gynecological evaluation | Negative | — |
| *Other* | | |
| 25-hydroxyvitamin D (nmol/L) | 22.81 deficient | >50 |

*CMV, cytomegalovirus; CK-MB, creatine kinase MB isoenzyme; dsDNA, double-stranded DNA; HSV-1, herpes simplex virus type 1; HIV, human immunodeficiency virus; TSH, thyroid-stimulating hormone. Arrows indicate direction of abnormality (↑ above reference range; ↓ below reference range).*

**Table S2. Electrodiagnostic findings (two concordant studies)**

| **Timepoint** | **Intervention** | **Clinical status** |
| --- | --- | --- |
| Day 0 (admission) | — | Severe DHS; unable to extend neck against gravity |
| Day 4 | MDT consultation; cranial-nerve signs identified; lumbar puncture performed | Tongue deviation and absent gag reflexes confirmed |
| Day 5 | IV dexamethasone 10 mg once daily initiated; rigid cervical collar; physiotherapy started | No clinical change yet |
| Day 9 | Continued dexamethasone | Able to lie supine without pillow support |
| Day 11 | Continued dexamethasone | Full active cervical extension in standing; walking with head erect |
| Day 12 | Dexamethasone discontinued; switched to oral prednisone 40 mg once daily | Complete symptomatic resolution |
| Day 14 | Discharge | Symptom-free |
| ~Day 19 | Prednisone discontinued without tapering | Symptom-free |
| Month 12 | Outpatient follow-up; no further immunosuppression | Complete sustained remission; no relapse |

*DHS, dropped head syndrome; IV, intravenous; MDT, multidisciplinary team.*

**Table S3. Treatment timeline and clinical response.**

| **Modality** | **Finding** |
| --- | --- |
| Motor nerve conduction (median, ulnar, radial, tibial, peroneal; bilateral) | Normal distal latencies, amplitudes and conduction velocities |
| Sensory nerve conduction (median, ulnar, radial, sural, superficial peroneal, saphenous, plantar; bilateral) | Normal |
| F-waves (median, ulnar, tibial; bilateral) | Normal latencies and persistence |
| Sympathetic skin response (four limbs) | Normal |
| Needle EMG, spontaneous activity | No fibrillation potentials or positive sharp waves in any muscle |
| Needle EMG, bilateral C5–C6 paraspinal muscles | Short-duration motor-unit potentials with early recruitment |
| Needle EMG, left triceps, extensor digitorum communis and rectus femoris | Partial short-duration motor-unit potentials; clinically silent |
| Needle EMG, other limb muscles | Normal |
| Interpretation | Pseudomyopathic (short-duration, early-recruitment) pattern without active denervation; repetitive nerve stimulation was not performed |

CMAP, compound muscle action potential; EMG, electromyography.

**Supplementary Figure S1.**

**Full-spine standing radiographs on admission.** (A) Posteroanterior projection and (B) lateral projection demonstrating cervical kyphosis with anterior translation of the head and mild left-sided cervicothoracic scoliosis, without significant thoracic or lumbar deformity. No osseous destructive lesion, vertebral body collapse, or paravertebral mass is identified. The global sagittal alignment is otherwise preserved, with the axis of the head projecting anterior to the sacrum secondary to the cervical deformity. “L” indicates the patient’s left side. The image file (Supplementary_Figure_S1.tif) is provided as a separate submission file.
